# S1P-Induced TNF-α and IL-6 Release from PBMCs Exacerbates Lung Cancer-Associated Inflammation

**DOI:** 10.3390/cells11162524

**Published:** 2022-08-15

**Authors:** Michela Terlizzi, Chiara Colarusso, Pasquale Somma, Ilaria De Rosa, Luigi Panico, Aldo Pinto, Rosalinda Sorrentino

**Affiliations:** 1 Department of Pharmacy (DIFARMA), University of Salerno, 84084 Salerno, Italy; 2 Anatomy and Pathology Unit, Ospedale dei Colli, AORN, “Monaldi”, 84131 Naples, Italy

**Keywords:** sphingosine-1-phosphate (S1P), lung inflammation, lung cancer

## Abstract

Sphingosine-1-phosphate (S1P) is involved in inflammatory signaling/s associated with the development of respiratory disorders, including cancer. However, the underlying mechanism/s are still elusive. The aim of this study was to investigate the role of S1P on circulating blood cells obtained from healthy volunteers and non-small cell lung cancer (NSCLC) patients. To pursue our goal, peripheral blood mononuclear cells (PBMCs) were isolated and stimulated with S1P. We found that the administration of S1P did not induce healthy PBMCs to release pro-inflammatory cytokines. In sharp contrast, S1P significantly increased the levels of TNF-α and IL-6 from lung cancer-derived PBMCs. This effect was S1P receptor 3 (S1PR3)-dependent. The pharmacological blockade of ceramidase and sphingosine kinases (SPHKs), key enzymes for S1P synthesis, completely reduced the release of both TNF-α and IL-6 after S1P addition on lung cancer-derived PBMCs. Interestingly, S1P-induced IL-6, but not TNF-α, release from lung cancer-derived PBMCs was mTOR- and K-Ras-dependent, while NF-κB was not involved. These data identify S1P as a bioactive lipid mediator in a chronic inflammation-driven diseases such as NSCLC. In particular, the higher presence of S1P could orchestrate the cytokine milieu in NSCLC, highlighting S1P as a pro-tumor driver.

## 1. Introduction

The sphingosine-1-phosphate (S1P) is a bioactive lipid mediator that acts either as an intracellular second messenger or as a ligand for its membrane receptors (S1PRs), carrying out biological effects in both physiological and pathological conditions [1].

S1P is generated by ceramidase, an enzyme that converts ceramide into sphingosine, and then phosphorylated in S1P by sphingosine kinases (SPHKs), SPHK I and/or SPHK II [1]. The intracellularly generated S1P can be exported and act in a paracrine or autocrine fashion, interacting with its five G protein-coupled receptors (S1PR1-5) to affect various cellular processes [2]. In particular, the physiological release/activity of S1P is involved in cell differentiation and growth, cellular architecture, vascular integrity regulation, immune cell trafficking and response. Instead, the pathological synthesis, release and activity of S1P are involved in angiogenesis and migration, likely exacerbating inflammatory disorders. An imbalance of the ceramide/S1P rheostat in favor of S1P is responsible for massive S1P synthesis, which is associated with the development of respiratory diseases, including cancer [3]. However, many doubts about the precise pathological role of S1P still need to be evaded.

In our recent study, we demonstrated that the activation of Toll-like receptor 9 (TLR9) induced the synthesis of endogenous S1P in lung adenocarcinoma cells [4]. The endogenous S1P induced by TLR9 boosted the release of pro-inflammatory cytokines that, in the context of the lung tumor microenvironment, foster tumor proliferation. Indeed, the activation of cell surface receptors and the nuclear S1P receptor 3/sphingosine kinase II (S1PR3/SPHK II) axis facilitated tumor cell proliferation [5], leading us to suppose that S1P could be at the basis of lung carcinogenesis associated with inflammatory patterns, as in the case of smoking exposure. Smoking mice as well as smoking patients with non-small cell lung cancer (NSCLC) and murine lungs of carcinogen-induced tumors were characterized by high levels of S1P associated with both an inflammatory pattern and tumor growth [5]. In support of this, others have demonstrated the S1P pro-survival role in cancer [6,7], leading them to suggest it as a biomarker and therapeutic target for various types of solid tumors [8,9]. It is well-known that fingolimod, a false substrate of SPHK I/II, has been approved in multiple sclerosis therapy, implying the potential therapeutic role of S1P [10]. This hypothesis is supported not only by the capability of S1P to induce tumor growth and metastasis, but also by its relevance to modulating the immunophenotype in the tumor microenvironment, orchestrating cancer progression and chemoresistance [11]. However, the molecular and cellular mechanisms are still unknown. Therefore, in the attempt to understand the effect of S1P, highly released from cancer cells, on the circulating cells, we used peripheral blood mononuclear cells (PBMCs) of lung cancer patients. We found that S1P exacerbated the pro-inflammatory milieu inducing both TNF-α and IL-6 release in a S1PR3-dependent manner with underlying activation of K-Ras and mTOR signaling.

## 2. Materials and Methods

### 2.1. Human Samples

Healthy volunteers (H) and lung cancer patients (LK) were recruited at the “Monaldi-Azienda Ospedaliera (AORN)-Ospedale dei Colli” Hospital in Naples, Italy, according to the Review Board which approved the project and the patients’ informed consent. The experimental protocol was performed in accordance with the guidelines and regulations provided by the Ethical Committee of the hospital (protocol no. 1254/2014). The healthy volunteers’ group (*n* = 29) did not have any pathologies or haematological alterations. The lung cancer patients’ group (*n* = 54) consisted of a comparable number of male and female patients diagnosed with adenocarcinoma or squamous cell carcinoma. The age of enrolled volunteers and patients had a mean of 50 ± 10 years old. Blood samples were collected and used within 24 h. PBMCs were isolated as described below.

### 2.2. Isolation and Treatment of Human PBMCs

PBMCs were isolated according to Ficoll’s protocol as already reported [12] and cultured in RPMI cell medium (supplemented with 1% Penicillin-Streptomycin and 10% Fetal Bovine Serum) in an atmosphere of 5% CO_2_ at 37 °C, seeded (7.5 × 10^4^ per well) and treated for 8 h. PBMCs were incubated with sphingosine-1-phosphate (S1P, 10 nM; #S9666; Sigma-Aldrich, Merck Life Science S.r.l., Milan, Italy). The concentration of S1P was chosen according to our preliminary data. We tested a concentration-dependent curve of S1P (0.1 nM up to 3 µM) and evaluated cytokine release. We found that the concentration-dependent administration of S1P described a bell-shaped curve, showing that the optimal concentration of S1P was 10 nM.

In another set of experiments, PBMCs were also treated with ceramidase inhibitor (D-NMAPPD, 5 µM; #SML2358; Sigma-Aldrich, Merck Life Science S.r.l., Milan, Italy), TY52156, a S1PR3 antagonist (αS1PR3, #5328; 10 µM; Tocris Bioscience, Ellisville, MO, USA), SKI II, a selective inhibitor of sphingosine kinases (SKI II, 10 µM; #2097; Tocris Bioscience, Ellisville, MO, USA), PF-543, a sphingosine-competitive inhibitor of sphingosine kinase I, SPHK I (PF543, 2 µM; #57177; Selleck Chemical, Houston, TX, USA), ABC294640 (Opaganib), a selective inhibitor of sphingosine kinase II, SPHK II (Opa, 60 µM; #915385-81-8; RedHill Biopharma, Tel-Aviv, Israel), FTI-276, a K-Ras inhibitor (FTI, 2 µg/mL; #F9553; Sigma-Aldrich, Merck Life Science S.r.l., Milan, Italy), Rapamycin, a potent and specific mTOR inhibitor (Rap, 100 ng/mL; #553210; Calbiochem, Sigma-Aldrich, Merck Life Science S.r.l., Milan, Italy), MG132, a proteasome inhibitor (MG132, 10 µM; #M8699; Sigma-Aldrich, Merck Life Science S.r.l., Milan, Italy). The concentration of all substances was chosen on the basis of the existing literature and on our previous experiments/data [4,5].

### 2.3. Cytokine Measurements

TNF-α and IL-6 were measured in cell-free supernatants, obtained after 8 h of cell treatment, by means of a commercially available enzyme-linked immunosorbent assay kit (ELISAs) (Diaclone SAS, Besançon, France). The absorbance wavelength was 450 nm. Cytokine levels were expressed as pg/mL.

### 2.4. Western Blotting Analysis

The expression of S1PR3 (55–70 kDa; diluted 1:500 in a PBS 1× solution containing 5%BSA; #ASR-013; Alomone labs; Jerusalem, Israel) and of ceramidase active form (40 kDa; N-acylsphingosine amidohydrolase 1, ASAH1; diluted 1:750 in a PBS 1× solution containing 2% BSA; #E-AB-10959; Elabscience, Houston, TX, USA) were evaluated in healthy volunteers (H)- and lung cancer (LK)-derived PBMCs. Furthermore, ceramidase, phospho-NF-κB p65 (p-p65; 65 kDa; diluted 1:500 in a PBS 1× solution containing 5% BSA; #SC-372; Santa Cruz Biotecnology, Inc.; Santa Cruz, CA, USA), phospho-IkBα (p-IkBα; 40 kDa; diluted 1:500 in a PBS 1× solution containing 5%BSA; #SC-8404; Santa Cruz Biotecnology, Inc.; USA), phopho-Erk (p-Erk; 44 kDa; diluted 1:1000 in a PBS 1× solution containing 5% BSA; #9101; Cell Signaling Technology, Inc., Danvers, MA, USA) and phospho-Akt (p-Akt; 60kDa; diluted 1:500 in a PBS 1 x solution containing 5%BSA; #9271S; Cell Signaling Tecnology, Inc.; Danvers, MA, USA) expression were evaluated after one hour treatment of PBMCs with S1P (10 nM). Glyceraldehyde-3-Phosphate Dehydrogenase (GAPDH; diluted 1:2000 in a PBS 1× solution containing 5%BSA; #TA890003; OriGene Technologies, Rockville, MD, USA) was used as loading control. Data were analysed by means of ImageJ software 1.53a http://imagej.nih.gov/ij (NIH, Bethesda, MD, USA). 

### 2.5. Statistical Analysis

Data are reported as median and represented as scatter dot plots. Statistical differences were assessed with two-tailed Wilcoxon matched-pairs signed rank test. *p* values less than 0.05 were considered significant. The statistical analysis was performed by using GraphPad prism 9.3.0 version (San Diego, CA, USA).

## 3. Results

### 3.1. S1P Induced the Release of Pro-Inflammatory Cytokines from Lung Cancer but Not from Healthy-Derived PBMCs

**Figure 1:**
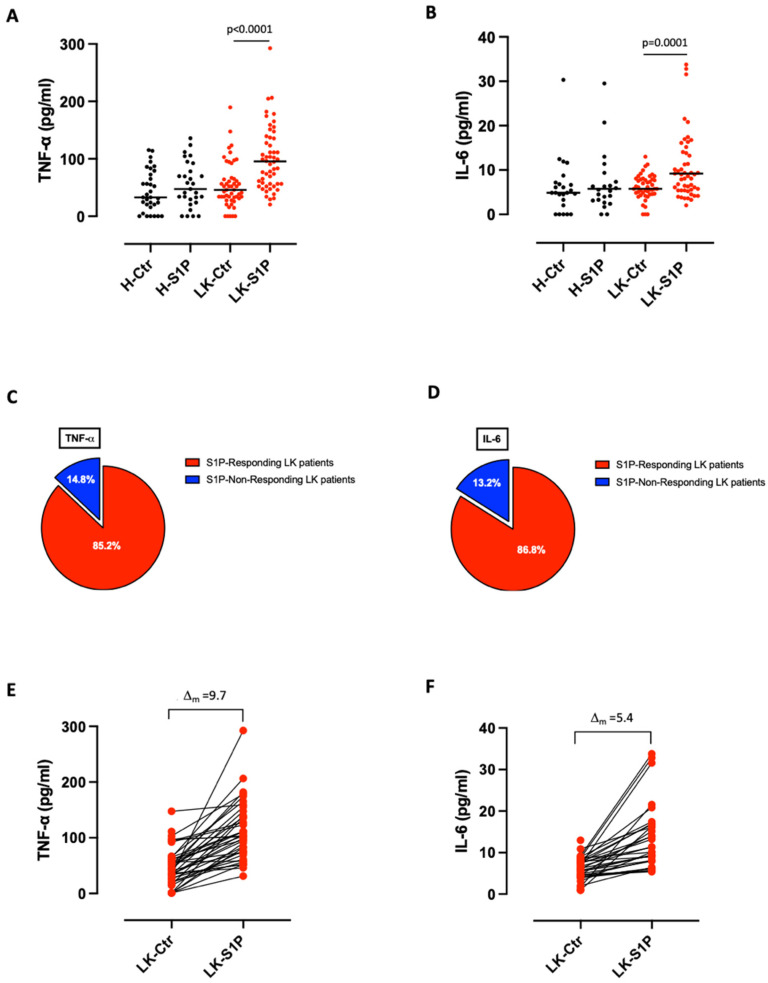
S1P induced the release of TNF-α and IL-6 from lung cancer-, but not from healthy-derived PBMCs. Healthy volunteers (H)- and lung cancer (LK)-derived PBMCs were stimulated with S1P (10 nM) and the release of TNF-α (**A**) and IL-6 (**B**) was evaluated after 8 h of treatment. *n* = 29 H and *n* = 54 LK were tested for TNF-α (**A**); *n* = 24 H and *n* = 53 LK were tested for IL-6 (**B**). (**C**) 85.2% of LK patients (red slice) were responsive to S1P treatment releasing TNF-α whereas, 14.8% (blue slice) of LK patients were not responsive to S1P stimulation. (**D**) 86.8% of LK patients (red slice) were responsive to S1P in terms of IL-6 release, whereas 13.2% (blue slice) of LK patients were not responsive. The mean delta (Δ_m_) of increment of the TNF-α levels after S1P stimulation was of 9.7 compared to controls (**E**). (**F**) The release of IL-6 from lung cancer-derived PBMCs after S1P addition showed a Δ_m_ of increment of 5.4 compared to controls. The Δ_m_ of increment was calculated as Δ_m_ = [(Cytokine levels after S1P addition-basal control)/basal control]/total number of patients. Basal control corresponded to non-stimulated cells. Data are represented as scatter dot plots indicating the median (confidence interval = 95%). Statistical differences were assessed by means of two-tailed Wilcoxon matched-pairs signed rank test.

An ever-growing body of evidence has now confirmed S1P involvement in inflammatory signaling(s) associated with the development of lung disorders [13], although the mechanism(s) is/are still elusive. Therefore, we stimulated PBMCs isolated from the blood of healthy volunteers (H) and lung cancer patients (LK) with S1P (10 nM), and the release of pro-inflammatory cytokines was evaluated. S1P treatment induced a significant increase of TNF-α and IL-6 release from LK-derived PBMCs, but not from H-derived PBMCs (Figure 1A,B). These data further support what has already been observed, that S1P promotes pro-inflammatory patterns in lung cancer but not in physiological conditions [4]. However, it has to be noted that not all PBMCs responded to S1P treatment in terms of TNF-α and IL-6 release: 85.2% (46 out of 54) of lung cancer patients responded to S1P treatment with the release of TNF-α (Figure 1C, red slice), while 86.8% (46 out of 53) with IL-6 release (Figure 1D, red slice). In particular, the mean delta (Δ_m_) of increment, in terms of cytokine release after S1P stimulation, was of 9.7 for TNF-α (Figure 1E) and of 5.4 for IL-6 (Figure 1F). In addition, we were not able to detect any increase in other inflammatory cytokines (e.g., IL-1α, IL-1β, IFN-α, IL-8 and IL-18) from both healthy and lung cancer-derived PBMCs after S1P stimulation (data not shown). We were only able to detect TNF-α and IL-6, implying that these two cytokines could be mainly involved in S1P-mediated inflammatory signaling in lung cancer-derived PBMCs.

### 3.2. The Inhibition of S1PR3 Reduced S1P-Induced TNF-α and IL-6 Release from PBMCs

**Figure 2:**
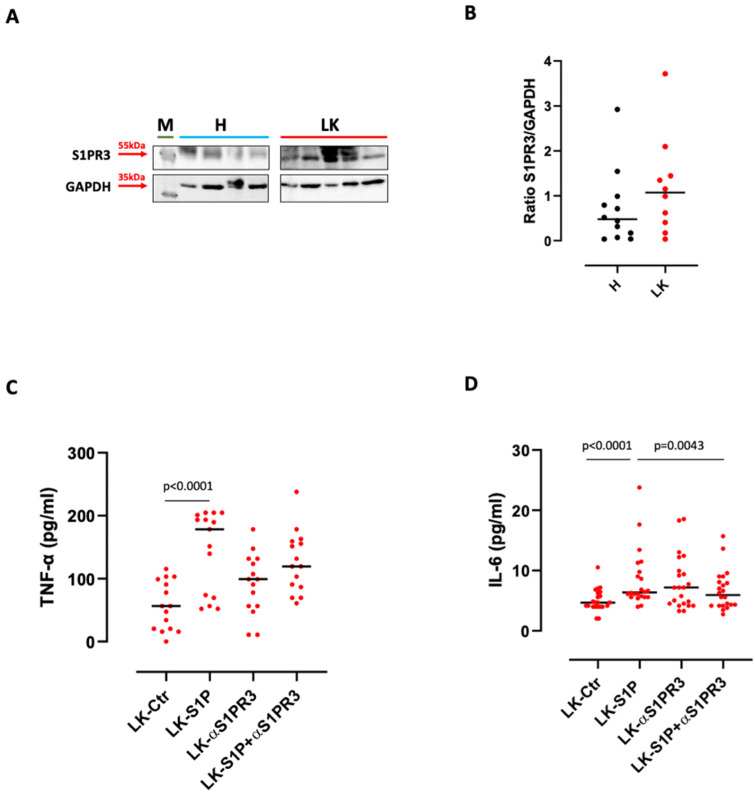
S1P-induced pro-inflammatory cytokine release was S1PR3 dependent. (**A**) Western blotting shows that healthy volunteers (H)-derived PBMCs expressed lower levels of S1PR3 compared to lung cancer (LK)-derived PBMCs. GAPDH was used as a loading control. The experiment was repeated three times. M refers to protein molecular weight marker. (**B**) S1PR3 expression analysis was performed by means of ImageJ software (NIH, USA) and expressed as a ratio between S1PR3 and GAPDH (loading control). S1PR3 inhibition with TY52156 (αS1PR3, 10 µM) reduced the release of TNF-α (**C**) and IL-6 (**D**) from LK-derived PBMCs after S1P (10 nM) addition. We used match-paired LK samples. *n* = 15 LK (Ctr-S1P) were tested for TNF-α (**C**) and *n* = 22 LK (Ctr-S1P) were tested for IL-6 (**D**). Data are represented as scatter dot plots indicating the median (confidence interval = 95%). Statistical differences were assessed by means of two-tailed Wilcoxon matched-pairs signed rank test.

Because S1P carries out its biological effect by interacting with its surface receptors (S1PRs) [1], we went on to evaluate their expression on H- and LK-derived PBMCs. S1PR3 was the most expressed receptor (Figure 2A), likewise previously observed on structural cells [5]. Whereas S1PR1 and S1PR2 were not detected on PBMCs (data not shown). Of note, S1PR3 was over-expressed on LK-isolated PBMCs compared to H-isolated PBMCs (Figure 2A,B). To understand whether S1P-induced pro-inflammatory cytokine release was dependent on this receptor, we treated PBMCs with a S1PR3 antagonist (αS1PR3, 10 µM) in the presence of S1P. The inhibition of S1PR3 led to an evident, but not statically significant, reduction of S1P-induced TNF-α (S1P median: 178.4 pg/mL vs S1P+αS1PR3 median: 119.5 pg/mL) (Figure 2C). Instead, S1P-derived IL-6 release was significantly reduced in the presence of an S1PR3 antagonist (Figure 2D), implying that the pro-inflammatory activity of S1P on PBMCs is mainly mediated by S1PR3.

### 3.3. S1P-Induced TNF-α and IL-6 Release from Lung Cancer-Derived PBMCs Was Ceramidase-Dependent

**Figure 3:**
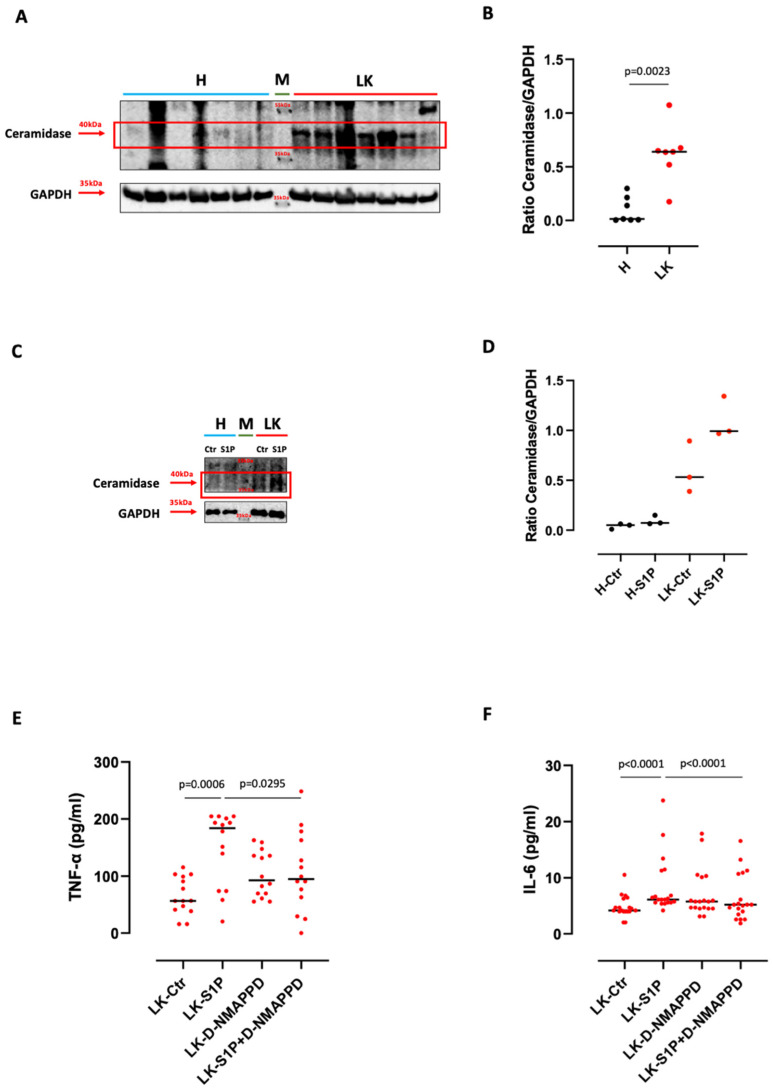
S1P-induced pro-inflammatory cytokine release was ceramidase dependent. (**A**) Western blotting shows that healthy volunteers (H)-derived PBMCs expressed lower levels of ceramidase active form (40 kDa) compared to lung cancer (LK)-derived PBMCs. GAPDH was used as a loading control. The experiment was repeated three times. M refers to the protein molecular weight marker. (**B**) Ceramidase active form expression analysis was performed by ImageJ software (NIH, USA) and expressed as a ratio between ceramidase and GAPDH. (**C**) One-hour S1P-stimulated LK-derived PBMCs over-expressed the ceramidase in its active form (40 kDa) compared to control (Ctr) and to H-isolated PBMCs. GAPDH was used as a loading control. The experiment was repeated three times. M refers to the protein molecular weight marker. (**D**) Ceramidase active form expression analysis was performed by ImageJ software (NIH, USA) and expressed as a ratio between ceramidase and GAPDH. Ceramidase inhibition by means of D-NMAPPD (5 µM) significantly reduced the release of TNF-α (**E**) and IL-6 (**F**) from LK-derived PBMCs, after S1P (10 nM) addition. *n* = 14 LK matched-pair samples were tested for TNF-α (**E**) and *n* = 19 matched-pair samples were tested for IL-6 (**F**). Data are represented as scatter dot plots indicating the median (confidence interval = 95%). Statistical differences were assessed by means of a two-tailed Wilcoxon matched-pairs signed rank test.

The ceramidase is an enzyme that converts ceramide into sphingosine, which is then phosphorylated into S1P by SPHK I/II [1]. Therefore, we moved on to evaluate the expression/activity of this enzyme. The ceramidase was over-expressed in its active form (40 kDa) in LK-derived PBMCs but not in H-derived PBMCs (Figure 3A,B), further confirming the involvement of the endogenous S1P in lung cancer. Interestingly, the stimulation of LK-derived PBMCs with S1P further increased the expression of the ceramidase in its active form, implying an increase in the endogenous production of S1P induced by the exogenous S1P via its interaction with S1PR3 (Figure 3C,D). This effect was not observed in healthy PBMCs (Figure 3C,D). To further support this hypothesis, we blocked the activity of the ceramidase by means of a powerful inhibitor, D-NMAPPD (5 µM). The pharmacological blockade of ceramidase in LK-derived PBMCs completely reduced the release of both TNF-α and IL-6 after S1P stimulation (Figure 3E,F), further confirming that the exogenous S1P boosts its own metabolism, favoring pro-inflammatory cytokines release from LK-derived PBMCs.

### 3.4. The Inhibition of SPHKs Reduced the Release of TNF-α and IL-6 after S1P Stimulation of Lung Cancer-Derived PBMCs

**Figure 4:**
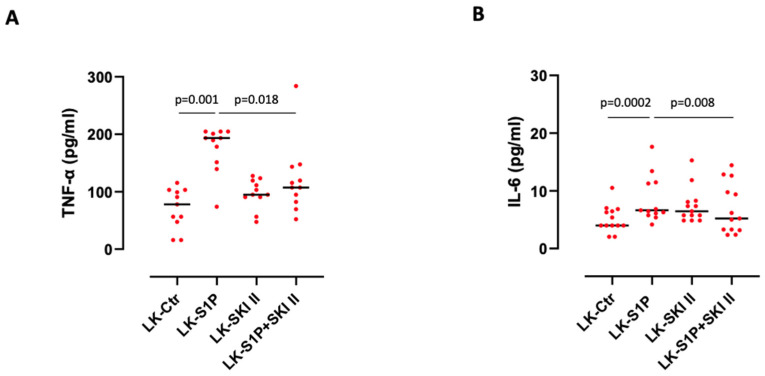

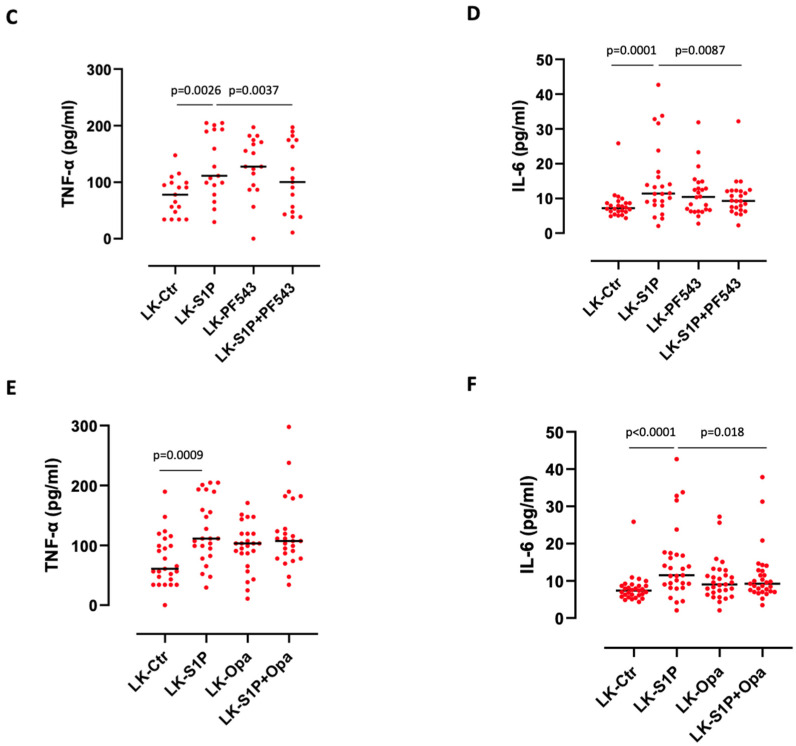
Sphingosine kinase inhibition reduced S1P-induced pro-inflammatory cytokine release. The inhibition of both SPHK I and II by means of SKI II (10 µM) reduced S1P-induced TNF-α ((**A**), *n* = 11) and IL-6 ((**B**), *n* = 13) release from lung cancer (LK)-derived matched-pairs PBMCs. The inhibition of SPHK I by means of PF543 (2 µM) reduced S1P-induced TNF-α ((**C**), *n* = 17) and IL-6 ((**D**), *n* = 24) release from LK-derived PBMCs. The inhibition of SPHK II by means of Opaganib (Opa, 60 µM) reduced S1P-induced IL-6 ((**F**), *n* = 29), but not TNF-α ((**E**), *n* = 25), from LK-derived PBMCs. Data are represented as scatter dot plots indicating the median (confidence interval = 95%). Statistical differences were assessed by means of a two-tailed Wilcoxon matched pairs signed rank test.

Sphingosine kinase I and II (SPHK I/II) are biological lipid kinases catalyzing ATP-dependent phosphorylation of sphingosine to S1P. SPHK I localizes mostly in the cytoplasm and migrates to the plasma membrane upon phosphorylation. It is involved in cancer cell proliferation and invasion and is correlated with severity and poor prognosis of cancers, and chemotherapy resistance [14]. SPHK II localizes mainly into the nucleus to inhibit DNA synthesis and regulate HDAC1/2 activity; downregulation of SPHK II reduces inflammation, proliferation and migration of tumor cells [15]. Here we evaluated whether SPHKs could be involved in the lung cancer-associated pro-inflammatory pathway mediated by S1P in PBMCs and which of the two was prevalent. The inhibition of both SPHK I and II, by means of the selective inhibitor SKI II (10 µM), completely abolished the release of TNF-α (Figure 4A) and IL-6 (Figure 4B) after S1P stimulation from LK-derived PBMCs compared to the treatment with the sole S1P. In another set of experiments to discriminate the role of SPHK I over SPHK II, we moved on to using two selective inhibitors, PF543 (2 µM) for SPHK I and Opaganib (Opa, 60 µM) for SPHK II. The inhibition of SPHK I, by PF543, significantly reduced the release of TNF-α (Figure 4C) and IL-6 (Figure 4D) after S1P addition, while the inhibition of SPHK II, by Opa, reduced S1P-induced IL-6 release (Figure 4F) but not of TNF-α (Figure 4E), shedding light on a different involvement of these two kinases in S1P-induced pro-inflammatory signaling. It is noteworthy that SPHK I inhibition in matched-pairs of samples significantly reduced S1P-induced TNF-α (S1P median: 111.4 pg/mL vs S1P+PF543 median: 100.3 pg/mL; *p* = 0.0037) (Figure 4C) and IL-6 (S1P median: 11.36 pg/mL vs S1P+PF543 median: 9.28 pg/mL; *p* = 0.0087) (Figure 4D). Instead, the inhibition of SPHK II in matched-pairs of samples by means of Opa solely reduced IL-6 (S1P median: 11.46 pg/mL vs S1P+Opa median: 9.19 pg/mL; *p* = 0.018) (Figure 4F). Together, these data imply that SPHKs are both involved in S1P-regulated pro-inflammatory signaling in LK-derived PBMCs, but SPHK II is predominant for IL-6 release.

### 3.5. S1P-Induced IL-6 Release from Lung Cancer-Derived PBMCs Was mTOR and K-Ras-Dependent

It is known that the interaction of S1P with its receptors activates oncogenic kinases, strengthening pro-survival signaling, implying the activation of PI3K/Akt/mTOR, NF-κB and/or K-Ras/Erk pathway [16,17]. Furthermore, these pathways can, in turn, regulate sphingolipid metabolism by inducing S1P synthesis [18]. To learn more about the mechanism underlying S1P-induced pro-inflammatory processes in the circulating cells of lung cancer patients, we moved on to investigate the involvement of these pathways.

**Figure 5:**
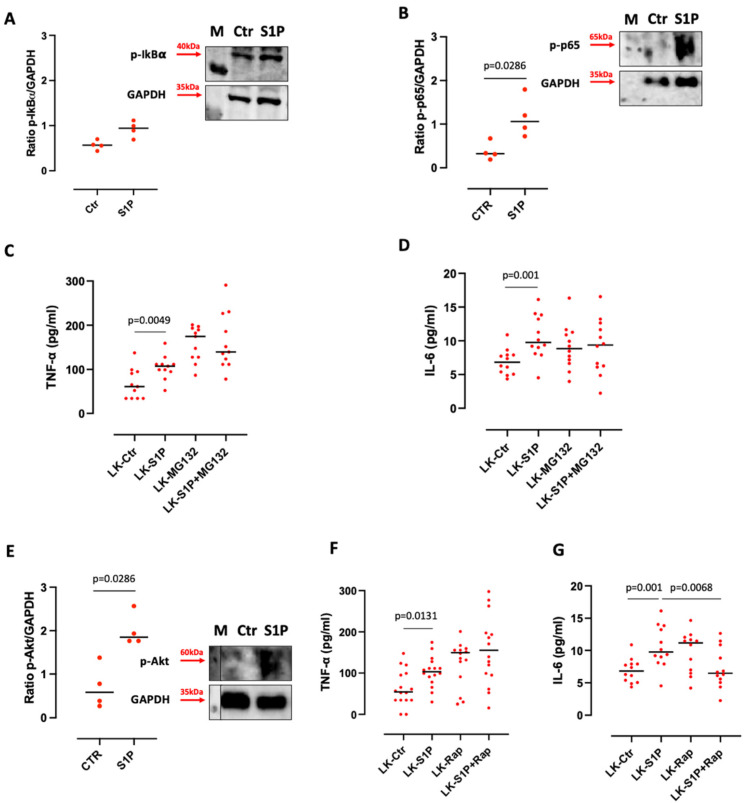

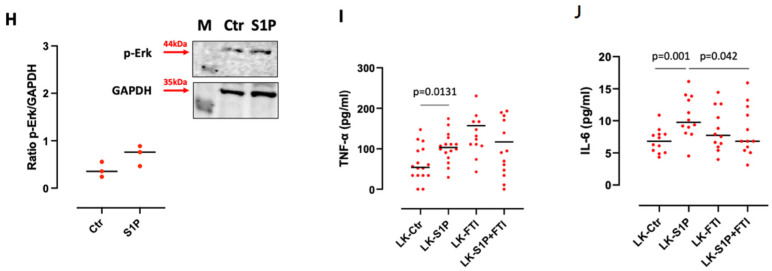
Akt/mTOR and K-Ras/Erk pathways were involved in the S1P-induced IL-6 release. One-hour S1P-treated lung cancer (LK)-derived PBMCs over-expressed phospho-IkBα (p-IkBα; 40kDa) (**A**) and phospho-NF-κB p65 (p-p65; 65kDa) (**B**) compared to control. GAPDH was used as loading control. M refers to the protein molecular weight marker. The experiment was repeated four times. NF-κB inhibition by means of MG132 (10 µM) did not reduce the release of TNF-α ((**C**), *n* = 11) and IL-6 ((**D**), *n* = 12) from LK-isolated PBMCs after S1P addition. (**E**) One-hour S1P-treated LK-derived PBMCs over-expressed phospho-Akt (p-Akt; 60kDa) compared to control. GAPDH was used as a loading protein. M refers to the protein molecular weight marker. The experiment was repeated four times. The inhibition of mTOR by means of Rapamicin (Rap, 100 ng/mL) reduced IL-6 ((**G**), *n* = 12), but not S1P-induced TNF-α release ((**F**), *n* = 16). (**H**) One-hour S1P-treated LK-derived PBMCs over-expressed phopho-Erk (p-Erk; 44 kDa) compared to control (Ctr). GAPDH was used as loading protein. M refers to protein molecular weight marker. The experiment was repeated three times. The inhibition of K-Ras by means of FTI-276 (FTI, 2 µg/mL) reduced IL-6 ((**J**), *n* = 12), but not S1P-induced TNF-α release ((**I**), *n* = 16). All samples were matched-pairs. Data are represented as scatter dot plots indicating the median (confidence interval = 95%). Statistical differences were assessed by means of two-tailed Wilcoxon matched-pairs signed rank test.

Although S1P was involved in the activation of NF-κB signaling, in that it was able to induce an overexpression of phospho-IkBα (Figure 5A) and phospho-NF-κB p65 (Figure 5B) in LK-derived matched-pairs of PBMCs, TNF-α (Figure 5C) and IL-6 (Figure 5D) release induced by S1P was not associated to this pathway, since the inhibition of NF-κB by MG132 did not affect their release. Thus, we went ahead and investigated other mechanisms potentially involved in S1P-induced pro-inflammatory cytokine release, modulating the PI3K/Akt/mTOR axis. After confirming the ability of S1P to potentiate this pathway in that it was able to induce the overexpression of phospho-Akt in LK-derived PBMCs compared to control (Figure 5E), we treated cells with Rapamycin, a potent and specific mTOR inhibitor (Rap, 100 ng/mL). TNF-α release was not reduced after S1P+Rap stimulation (Figure 5F), while IL-6 levels were significantly reduced when mTOR was inhibited in the presence of S1P compared to the sole S1P (Figure 5G). This led to suppose the involvement of Akt/mTOR axis in S1P-induced IL-6, but not of TNF-α release. Moreover, we evaluated the downstream K-Ras/Erk signaling in S1P-dependent pro-inflammatory cytokine release. We found that the stimulation of LK-derived PBMCs with S1P induced phospho-Erk overexpression (Figure 5H). Furthermore, the inhibition of K-Ras by means of FTI (2 µg/mL) significantly reduced S1P-induced IL-6 (Figure 5J), but not TNF-α release (Figure 5I).

Altogether, these data show that S1P-induced IL-6 release from LK-derived PBMCs is mTOR and K-Ras-dependent, differently than TNF-α, which release is likely regulated by other(s) mechanism(s), which need further investigation.

## 4. Discussion

**Figure 6:**
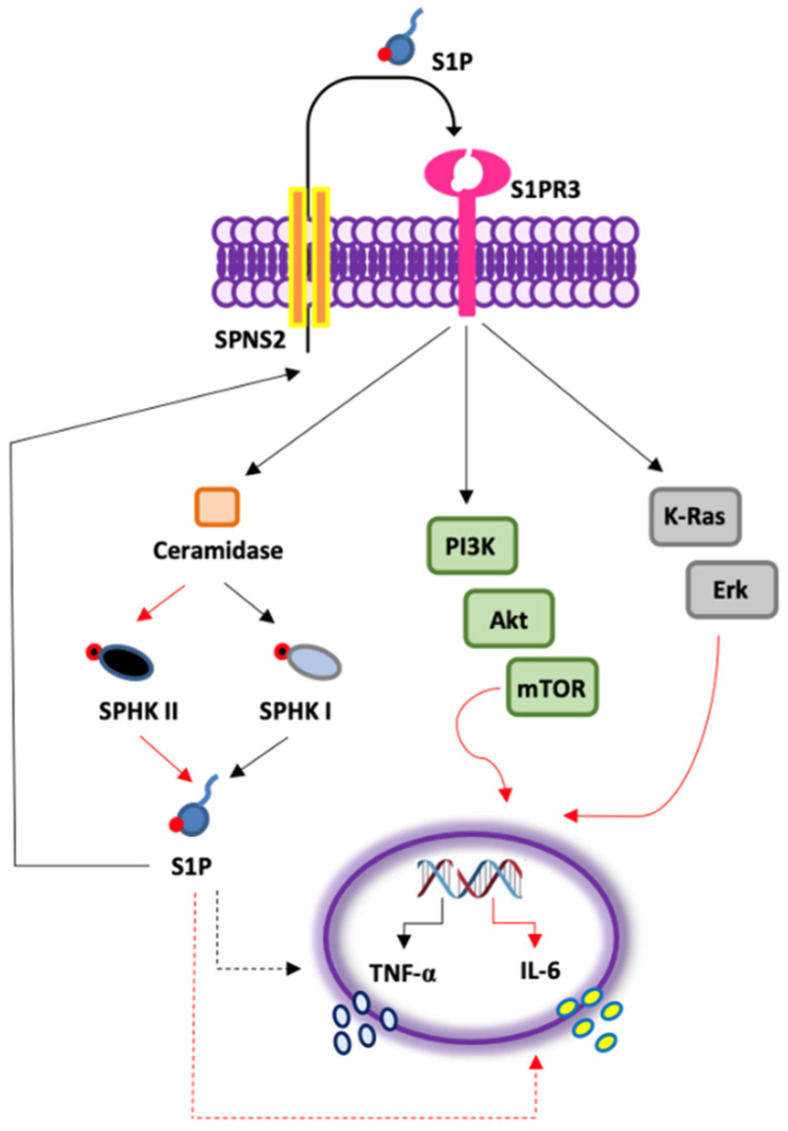
S1P exacerbates the pro-inflammatory milieu by inducing pro-inflammatory cytokine release from lung cancer-derived PBMCs in a S1PR3-dependent manner. The activation of S1PR3 by the exogenous S1P enhances its own production and fosters the release of TNF-α in a SPHK I-dependent manner (black arrows), and of IL-6 via SPHK I/II (red arrows); S1P-induced IL-6, but not TNF-α, release from PBMCs of lung cancer patients is mTOR- and K-Ras-dependent (red arrows).

The involvement of S1P in cancer is currently known [19], however, the underlying mechanism(s) of S1P-favored cancer establishment/progression and immunomodulation are still elusive. In this study, we found that:

1. S1P exacerbates the pro-inflammatory milieu by inducing IL-6 and TNF-α release from LK-derived PBMCs in a S1PR3-dependent manner;
2. The activation of S1PR3 by the exogenous S1P induces the release of TNF-α in a SPHK I-dependent manner, and of IL-6 via SPHK I/II;
3. S1P-induced IL-6, but not TNF-α, release from PBMCs of lung cancer patients is mTOR- and K-Ras-, but not NF-κB-dependent (Figure 6).

These results highlight a molecular mechanism exploited by S1P in circulating cells of lung cancer patients. In our previous studies, we found that lung cancer epithelial cells were responsive to exogenous S1P, but the major source of TNF-α was related to the activation of TLR9. In particular, TLR9 activation in lung cancer epithelial cells increased the release of TNF-α, but not of IL-6, through an imbalance of the ceramide/S1P rheostat in favor of S1P [4]. The activation of TLR9 led to the production of endogenous S1P, which, through an inside-out mode, favored TLR9/NF-κB-mediated TNF-α release via S1PR3 [4]. Here, instead, we found that S1P activity was related to S1PR3 on circulating cells of lung cancer patients but not by TLR9 (data not shown). The exogenous S1P interacted on S1PR3 and favored IL-6 and TNF-α release, implying a major role of circulating S1P on these cells. In support of this, it was proven that lung cancer patients have higher levels of circulating S1P compared to healthy subjects [20]. Therefore, it could be likely that S1P could orchestrate circulating cells toward a specific immunophenotype as well as toward an inflammatory signature that could influence tumor growth. Indeed, it is known that S1P released by cancerous cells functions in an autocrine or paracrine manner to protect cancer cells against apoptosis, favoring cancer cell growth and proliferation, angiogenesis and metastasis [19]. In support of this, we also demonstrated that adenocarcinoma cells were more susceptible to cell growth after S1P stimulation [5].

TNF-α and IL-6 are two key inflammatory cytokines linked to chronic inflammatory diseases and cancer [21]. A large body of literature points to NF-κB, originally described as a gatekeeper for inflammatory control of immune cell responses, as a key transcriptional factor in the regulatory network activated by these inflammatory cytokines. Recent studies suggest the involvement of the S1P/S1PR3 axis in NF-κB inflammatory response [22,23]. Despite S1P potentiates NF-κB activation in our experimental conditions, this pathway was not involved in S1P-induced TNF-α and IL-6 release from LK-derived PBMCs. Instead, our data show that S1P/S1PR3 axis leads to mTOR and K-Ras signaling that allow IL-6 release from LK-derived PBMCs. However, we were not able to define the molecular mechanism underlying TNF-α, which was not NF-κB, mTOR or K-Ras-dependent. Further studies are needed. 

Another important issue in this study is that the outer S1P can lead to the production of endogenous S1P in LK-derived PBMCs, increasing IL-6 release in a ceramidase, SPHK II-dependent manner. On the contrary, ceramidase and SPHK I were involved in TNF-α release. In support, Beckman and colleagues and others proved that ceramidase is able to induce oncogenic Akt/mTOR and K-Ras/Erk signaling in different types of cancers [17,24,25]. In this scenario, the pro-survival activity on immune cells [26] and the release of the immunosuppressive IL-6 in tumor microenvironment once recruited to the tumor site, could exacerbate tumor progression and likely explain therapy resistance. Indeed, many studies correlate high levels of IL-6 in the blood of lung cancer patients with poor prognosis and drug resistance, although the mechanism is still unclear [27,28,29]. We identify a hitherto unknown S1P/S1PR3 axis involved in the amplification of PBMC-driven inflammation through IL-6 release in lung cancer, likely explaining the involvement of S1P in lung cancer. In particular, the activation of S1PR3 on lung cancer-derived PBMCs by the exogenous S1P enhances its own metabolism and fosters the release of TNF-α in a SPHK I-dependent manner (Figure 6, black arrows), and of IL-6 via SPHK I/II (Figure 6, red arrows); S1P-induced IL-6, but not TNF-α, is mTOR- and K-Ras-dependent (Figure 6, red arrows).

## 5. Conclusions

Our findings open up new scenarios on the physiological and pathological role of S1P and shed light on a novel and never investigated signaling network between lung oncogenesis and lung cancer-associated inflammation in a S1P-dependent manner. We believe that the identification of S1P-induced inflammatory pathways in circulating cells of lung cancer patients together with its role in structural tumor cells can open new perspectives for drug discovery for lung cancer patients.

## Data Availability

Not applicable.
